# Oral Release Behavior of Wine Aroma Compounds by Using In-Mouth Headspace Sorptive Extraction (HSSE) Method

**DOI:** 10.3390/foods10020415

**Published:** 2021-02-13

**Authors:** María Pérez-Jiménez, Carolina Muñoz-González, María Angeles Pozo-Bayón

**Affiliations:** Instituto de Investigación en Ciencias de la Alimentación (CIAL), CSIC-UAM, C/Nicolás Cabrera, 9, 28049 Madrid, Spain; maria.perez@csic.es (M.P.-J.); c.munoz@csic.es (C.M.-G.)

**Keywords:** wine aroma, oral release, aroma persistence, in-mouth headspace sorptive extraction

## Abstract

The oral release behavior of wine aroma compounds was determined by using an in-mouth headspace sorptive extraction (HSSE) procedure. For this, 32 volunteers rinsed their mouths with a red wine. Aroma release was monitored at three time points (immediately, 60 s, and 120 s) after wine expectoration. Twenty-two aroma compounds belonging to different chemical classes were identified in the mouth. Despite the large inter-individual differences, some interesting trends in oral release behavior were observed depending on the chemical family. In general, esters and linear alcohols showed rapid losses in the mouth over the three sampling times and therefore showed a low oral aroma persistence. On the contrary, terpenes, lactones, and C_13_ norisoprenoids showed lower variations in oral aroma release over time, thus showing a higher oral aroma persistence. Additionally, and despite their low polarity, furanic acids and guaiacol showed the highest oral aroma persistence. This work represents the first large study regarding in-mouth aroma release behavior after wine tasting, using real wines, and it confirmed that oral release behavior does not only depend on the physicochemical properties of aroma compounds but also on other features, such as the molecular structure and probably, on the characteristics and composition of the oral environment.

## 1. Introduction

Wine odorant molecules belong to very different chemical classes (esters, alcohols, aldehydes, terpenes, phenols, etc.) in which their physicochemical properties such as volatility, boiling point, polarity, hydrophobicity, and/or molecular structure all differ.

The different physicochemical properties of odorant molecules also determine their release from the wine matrix and therefore their transfer to the surrounding air that will carry these volatile chemical molecules to the olfactory receptors when breathing. This is the orthonasal route and it is the main reason for the perceived odor when we smell a wine. However, during wine tasting, odorant molecules are released in the oropharyngeal cavities, and the expiration flows carry them to the olfactory receptors by the so-called retronasal pathway. In this case, as well as the physicochemical characteristics of the odorant molecules and wine matrix composition, factors related to the individual oral physiology (breathing flow, dilution with saliva, interaction of odorants with salivary proteins, biochemical transformation by salivary enzymes, etc.) can also affect aroma release [1,2,3,4,5]. Moreover, the formation of wine residues or the adsorption of odorant molecules in the oral mucosa might be the origin of aroma reservoirs ready to be released in the successive swallowing–exhalation episodes of the remaining saliva in the mouth once the wine has been swallowed [6]. This is the origin of the long lasting aroma perception, also known as after-odor or aroma persistence [7].

The immediate and prolonged retronasal aroma perception is of great importance, since it is a key factor in determining wine quality and ultimately consumer preferences [8]. Because of this, in the last couple of decades, the study of the retronasal aroma of wine has gained popularity and the number of scientific works dealing with this topic has increased. Most of these studies have been carried out using sensory analysis [9] and using in vitro headspace experiments simulating wine oral conditions [10,11,12,13]. More recently, the use of in vivo approaches to monitor retronasal aroma release in more realistic wine consumption situations have been used, although these works are still scarce [7,14,15,16,17,18,19,20].

Both types of approaches can provide us with information about the behavior of wine odorant compounds during real or simulated wine consumption conditions that might better correlate to wine aroma perception than when only using the volatile profile analysis of a wine. In this sense, previous works have attempted to study this behavior by using static or dynamic headspace analysis [10,11,21] to simulate oral conditions. From these studies, it was shown that saliva differently affected the rate of aroma release depending on the type of aroma compound and on the wine matrix composition. More recently, Piombino and co-workers (2019) [13] showed that the release of volatile compounds from wine was strongly related to hydrophobicity. While aroma compounds with logP < 0 increased their retronasal release, odorants with 2 < logP < 5 showed an opposite trend, and aroma compounds with 0 < logP < 2 were the most affected compounds by the wine matrix components as residual sugars.

Although very valuable, in vitro oral conditions do not perfectly represent the physiological conditions of the oral cavity. Different works have found that saliva enzymes and oral microbiota can affect aroma compounds in the mouth in a different way depending on the individual, which in turn could be difficult to mimic by only using in vitro conditions. For instance, salivary enzymes are able to hydrolyze odorless glycosylated precursors [22,23] or metabolize odorants into their degradation products [4,24,25,26,27,28,29]—both cases give rise to different volatile odorant metabolites depending on individual differences in saliva composition.

In this sense, in vivo aroma analysis should be better suited to determine the retronasal release behavior of aroma compounds in the mouth during wine tasting. For instance, Esteban-Fernández et al. (2016) [15] used in vivo intraoral Solid Phase Microextraction (SPME) to compare the retronasal release behavior of six aroma compounds (isoamyl acetate, ethyl hexanoate, linalool, guaiacol, β-phenylethanol, and β-ionone) in the mouth of three individuals after tasting aromatised wines. They confirmed the impact of compound hydrophobicity on the degree of adsorption to oral mucosa, which provoked differences in in-mouth aroma release kinetics in the post-ingestion phase.

These works have a great scientific value since they represent the first analytical studies performed in real wine consumption conditions to monitor retronasal aroma release. However, because of the relatively low number of individuals employed to perform these assays, it would be interesting to set up new studies using a representative number of volunteers and using real wines, which will allow us to obtain more straightforward conclusions on the behavior of wine odorants in the mouth.

To do so, we utilized the in-mouth headspace sorptive extraction (HSSE) technique, which has been proven to be a reliable and feasible tool that allows for the monitoring of oral aroma release of a great number of different odorant chemical classes at real wine concentrations [18]. This methodology is based on the application of a polydimethyl siloxane (PDMS) twister in the mouth to perform the headspace intra-oral aroma extraction after wine intake. The twisters are further desorbed and analyzed by Gas Chromatography-Mass Spectrometry (GC-MS). One of the main advantages of this technique is that the twisters with the breathing aroma extract can be automatically desorbed in the thermal desorption unit (TDU) of the GC–MS, allowing for the analysis of a higher number of wine breath extracts and thus increasing the possibility of working with a greater number of individuals. Additionally, the lower in-mouth extraction times (30 s) compared to other in vivo methods [15] will allow us to perform more in-mouth aroma samplings in a shorter period of time (80 s) once the wine has been ingested.

In this frame, the aim of this work was to assess the oral release behavior of the naturally occurring wine aroma compounds, composed of different volatile chemical families at different concentrations, by using the in-mouth HSSE procedure. To do this, we instigated 32 volunteers to rinse their mouths with a red wine for 30 s. Subsequently, a glass device provided with a PDMS twister was placed in the mouth in order to monitor the aroma released at three different times (immediately, 60 s, and 120 s) after the wine was expectorated.

## 2. Materials and Methods

### 2.1. Wine Chemical Composition

A commercial red wine (Marqués de Murrieta, 2017) from the Tempranillo grape variety was employed in this study. The non-volatile composition of the wine: pH (4.0 ± 0.3), total polyphenol content (1917.3 ± 10.3 mg of gallic acid/L), free amino acids (561.2 ± 72.2 mg Leu/L), free amino acids plus peptides (328.9 ± 11.0 mg Leu/L), neutral polysaccharides (4014.1 ± 741.4 mg mannose/L), and procyanidins (1365.9 ± 42.0 mg catechin/L) was previously determined.

The volatile composition of the red wine was assessed using a headspace sorptive extraction method and gas chromatography analysis (HSSE-GC–MS) using a 20 mm length × 0.5 mm PDMS twister (Gerstel, Mülheim an der Ruhr Germany) as previously described [18]. For the quantification, a calibration curve for each aroma compound was carried out (Table S1) using the same conditions already described [18].

### 2.2. In-Mouth Aroma Analysis

#### 2.2.1. Subjects

Thirty-two subjects—15 females (47%) and 17 males (53%) between the ages of 18 and 72 years old—participated in this study. Fifty percent of the participants were younger than 35 years old and the other 50% were older than 50 years old. They did not have any known illnesses and they all had normal olfactory and gustatory functions. The participants were informed about the purpose of this study and they all gave their written consent to participate in this study. The project in which this study was enclosed was also approved by the Bioethical Committee of the Spanish Council of Research (CSIC).

#### 2.2.2. In-Mouth HSSE Procedure

**Figure 1:**
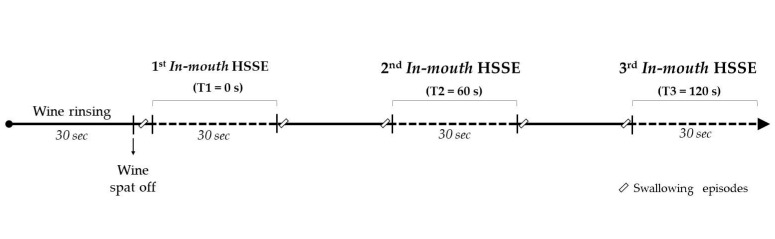
Sampling procedure followed for in-mouth HSSE aroma monitoring.

To determine both the immediate and the prolonged oral aroma release, we applied the previously published in-mouth HSSE method [18]. Briefly, the volunteers performed gentle rinses with the wine (15 mL) and 30 s after, they spat out the wine. During these rinses, swallowing episodes were not allowed and the lips and the velum tongue had to stay closed. For the intra-oral aroma extraction, a customized glass holder device (Segainvex-UAM, Madrid, Spain) provided with a 20 mm × 0.5 mm (length × film thickness) PDMS twister inside was employed. The volunteers placed the glass device in their mouths 5 s after spitting out the wines. To avoid any contact between the twister and the oral surfaces, we ensured that aroma sampling was performed in the headspace of the mouth. This procedure was repeated 3 times in order to determine the intra-oral aroma release at 3 different times after the wine expectoration. These sampling times were performed immediately (T1), 60 (T2) seconds, and 120 (T3) seconds after the wine rinses. For this, 3 different in-mouth devices with 3 different twisters were employed. Between each aroma monitoring, the subjects kept their lips closed, breathed normally through their nose, and were told to only swallow twice: immediately before introducing the twister into the mouth and immediately after removing it. Figure 1 shows a schematic representation of the main steps of the sampling procedure. Volunteers were previously trained in the in-mouth HSSE procedure before the wine sampling evaluation.

The twisters were removed from the glass device with a magnet bar and desorbed in a thermal desorption unit (TDU) (Gerstel) after the oral aroma sampling. Twenty different PDMS twisters were used for the whole experiment. In order to check their variability, we previously tested them all using a synthetic aromatized wine. Differences among the PDMS twisters in their aroma extraction were always lower than 5%.

Each analysis was performed 3 times for each volunteer (*n* = 32). Before the in-mouth aroma extraction and between wine replicates, volunteers washed their mouths by rinsing with a pectin-water solution (1 g/L). After rinses, they waited 15 min before starting a new in-mouth HSSE analysis.

#### 2.2.3. Thermal Desorption

The PDMS twisters were desorbed in the thermal desorption unit (TDU) coupled to a cooled injection system (CIS-4) (Gerstel). The TDU was configured in splitless mode. The TDU ramp temperature was first 40 °C, then increased at 60 °C/min to 240 °C and then held for 5 min. The CIS was employed for analyte cryofocusing by using liquid nitrogen. The CIS ramp temperature started at −100 °C, then heated to 240 °C at 12 °C/min, and held for 5 min. The injection was configured in solvent vent mode.

#### 2.2.4. GC–MS Analysis

Aroma analysis was carried out in a 6890 N GC coupled to a 5973 N mass spectrometer (Agilent). The stationary phase was a DB-WAX polar capillary column with dimensions of 30 m × 0.25 mm and film thickness of 0.50 μm (Agilent, J&W Scientific, Folsom, CA, USA), while the mobile phase was helium gas at a flow rate of 1 mL/min. The oven temperature conditions were the same as those previously described [18,30]. The oven started at 40 °C, then it raised to 130 °C at 4 °C/min, and finally it rose to 240 °C at 8 °C/min and kept for 5 min.

The temperatures of the MS system were configured in the following way: 270 °C for the transfer line, 150 °C for the quadrupole, and 230 °C for the ion source. The electron impact was fixed at 70 eV and the ionization current was fixed at 10 μA. Both selected ion mass monitoring (SIM) and full scan mode (mass range of 35–350 *m*/*z*) were used for data acquisition. The detected peaks were identified by comparing the mass spectra and the retention times with those of the same reference compounds analyzed with the same chromatographic conditions and by using the NIST 2.0 database.

As a result, the absolute peak areas (APAs) of the volatile compounds released in the mouth were obtained. APAs (3 repetitions) were used to compare the 3 in-mouth sampling points.

### 2.3. Statistical Analysis

For the statistical analysis, the XLSTAT 2020.4 software (Addinsoft, Paris, France) was employed. One-way ANOVA and Tukey test were applied to check for significant differences among APAs from the 3 in-mouth sampling points (T1, T2, and T3) and to check for mean comparison. A principal component analysis (PCA) was applied in order to examine how the physicochemical properties of the aroma compounds influenced their oral release in each sampling time. A significance level of α = 0.05 was fixed in all the statistical analyses.

## 3. Results and Discussion

### 3.1. Oral Aroma Release Behavior after Wine Tasting

To determine the oral aroma release behavior after wine consumption, we applied the in-mouth HSSE procedure [18]. For this, 32 volunteers rinsed their mouths with a red wine for 30 s and then spat it out. A PDMS twister was then placed inside the mouth to monitor aroma release for 30 s (T1). This procedure was also repeated for 60 s and 120 s (T2 and T3, respectively) after wine expectoration, as shown in Figure 1. Despite the large dilution of the aroma compounds in the mouth due to the exhalation flows following wine expectoration [31,32], all the aroma compounds detected in the headspace of the wine (Table S1) were also identified in the mouth of the volunteers (Table 1 and Figure S1). In order to check if there were significant differences (*p* < 0.05) in aroma release through the three monitoring times, we performed one-way ANOVA. Table 1 shows these results.

As it can be seen in Table 1, from the 22 compounds identified in the mouth, 12 of them showed significant variations (*p* < 0.05) depending on the sampling point (T1, T2, T3). Esters and alcohols were the most affected chemical groups by the sampling time. All of them, except ethyl butanoate, ethyl pentanoate, and Z-3-Hexen-1-ol, showed significant differences (*p* < 0.05) in the amount released depending on the sampling point. The fast decrease of esters and alcohols in the oral cavity as a function of time revealed that both groups of compounds showed a relatively low oral persistence. In addition to them, the compounds β-ionone and γ-nonalactone also showed significant differences (*p* < 0.05) depending on the sampling point. Contrarily, other volatile compounds belonging to other chemical families, such as furanic acids, terpenes, or the volatile phenol guaiacol, did not show significant differences depending on the sampling point, suggesting that they remained in the oral cavity for longer times. Both the chemical structure of the aroma compounds and their physicochemical properties (also reported in Table 1) might be responsible for the differences in the aroma release behavior.

**Figure 2:**
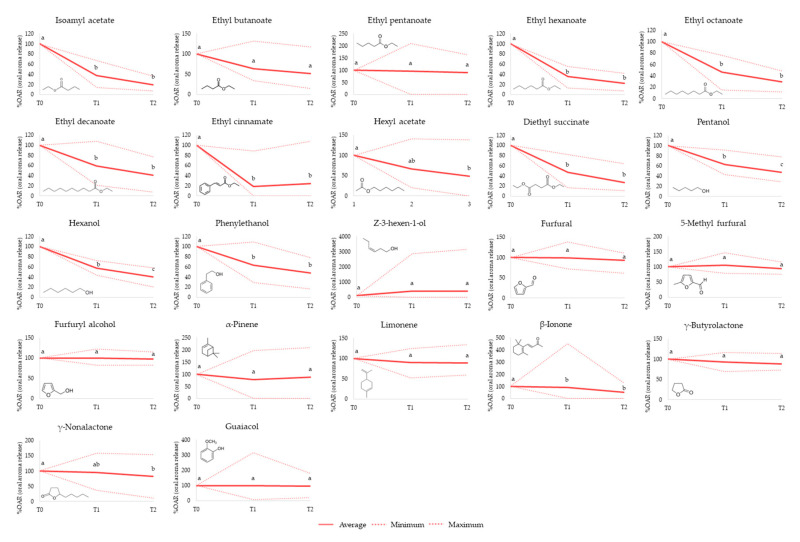
Oral aroma release profiles (%OAR) of 22 wine aroma compounds at 0 (T1), 60 (T2), and 120 (T3) seconds after of wine expectoration. The solid line shows the average value, while the dotted lines show the maximum and minimum values from three repetitions from the same wine and 32 volunteers (*n* = 96). Different letters above the points denote different significance level (*p* < 0.05) from the Tukey test.

Therefore, in order to further investigate the in-mouth aroma release behavior of the different chemical groups, we calculated the percentage of oral aroma release (%OAR) at each sampling point. For this, the amount of aroma released in the first sampling point, immediately after wine expectoration (T1), was considered as 100% (%OARt1). From this, the percentage of aroma release after 60 s (%OARt2) and 120 s (%OARt3) was also calculated. Figure 2 shows these results together with the chemical structure of each aroma compound. It also depicts the average, minimum, and maximum %OAR values corresponding to three repetitions of the same wine tested for the 32 volunteers for each sampling point (*n* = 96).

As it can be seen (Figure 2), very wide minimum and maximum %OAR intervals were observed for most wine volatile compounds. This was expected considering the large in-mouth aroma release variations that can be explained by differences in oral (breathing flows, saliva composition, oral cavity volumes) or other physiological features (age, gender), which have been related to significant differences in aroma release during food and beverage consumption [2,37,38]. Other factors related to variations from the instrumental methodology would be unlikely since all the participants were previously trained in the in-mouth HSSE procedure and the repeatability of this procedure was previously proven to be lower than 18% for a large number of different types of wine volatiles [18].

As can be seen in Figure 2, except for ethyl pentanoate, the rest of the esters showed a similar oral release behavior. This was characterized by a progressive reduction in oral aroma release over time, with the largest aroma losses (between 50 to 80%) in the third sampling time (T3) (Figure 2), which was 120 s after wine rinsing. Isoamyl acetate and ethyl hexanoate showed the lowest oral release in the third sampling point (T3), with %OAR of 19% and 22%, respectively (Figure 2). Thus, these esters exhibited the lowest oral persistence. These results are in agreement with the results from a previous work using intra-oral SPME approach and aromatized wines [15] and also confirmed the congruence between both in-mouth extraction procedures. Some reasons, such as the weaker interactions of these compounds to oral components (salivary proteins, oral mucosa cells) or the degradation of esters by salivary esterase enzymes might contribute to explaining the rapid loss of these compounds in the mouth [24,27,29,39]. However, as shown in Figure 2, some differences in the oral release behavior among the different esters were also found. For instance, in the case of ethyl decanoate (logP = 4.79), the most hydrophobic assayed linear ester, the %OARt3 was 41%, while in the case of shorter and less hydrophobic linear esters such as ethyl octanoate (logP = 3.81) and ethyl hexanoate (logP = 2.83), the percentages of %OARt3 were 29% and 22%, respectively. These results showed that 120 s after wine expectoration, the oral amount of ethyl decanoate was almost double that determined by the other two esters. These results are in agreement with those reported by Muñoz-González and co-authors (2019) [20], who also observed a higher persistence of ethyl decanoate compared to ethyl hexanoate and isoamyl acetate by using in-nose Proton Transfer Reaction-Mass spectrometry (PTR–MS). On the contrary, some esters with lower molecular weight, such as ethyl butanoate and ethyl pentanoate, did not significantly decrease over time. This could be mostly due to the large individual variations observed among volunteers. Thus, while some volunteers showed a decrease in the oral release of these esters over time, others showed an increase in their oral release. This dissimilar behavior among volunteers deserves further attention.

The linear alcohols (pentanol and hexanol) showed a similar behavior to that previously found for esters (Figure 2), which was characterized by a progressive decrease in their oral release over the three in-mouth aroma extractions (Figure 2). As shown in Figure 2, up to 40–50% of the initial aroma content remained 120 s after wine expectoration (%OARt3) (Figure 2). On the basis of these results, we can consider these alcohols as compounds with a relatively low-medium oral persistence. Although alcohols have not been reported to be metabolized by salivary enzymes [4,25], their low-medium oral persistence could be due to their weak interaction with oral physiology [15]. Interestingly, the linear alcohol cis-hexen-1-ol did not follow the same trend as the other alcohols. Not only did the release of this compound not decrease, but it increased in the second and third sampling points (Figure 2). This atypical behavior could be linked to the de novo formation of this metabolite from wine aroma precursors by oral microbiota or salivary enzymes. In fact, previous in vitro studies have proven the formation of cis-3-hexen-1-ol from glycosylated wine aroma precursors by isolated oral bacteria from saliva [23]. However, new experiments should be addressed in order to confirm these results. Regarding the cyclic alcohol phenylethanol, although a decrease in %OAR over time was observed, there were no differences between the percentage of aroma released 60 and 120 s after wine expectoration. This percentage was about 50% of the initial amount of this compound in the mouth, confirming its higher oral persistence compared to the linear alcohols, which was in agreement with results from a previous work using intra-oral SPME [15].

On the other hand, furanic acids (furfural logP = 0.83, 5-methyl furfural logP = 0.67, and furfuryl alcohol logP = 0.45) and the volatile phenol guaiacol (logP = 1.34) showed very little changes (<5%) in their release over the three sampling times (Figure 2). Thus, these compounds were still present in the mouth 120 s after wine expectoration, indicating a relatively high oral persistence. These results are in agreement with the high oral adsorption determined for guaiacol after the application of the spit off odorant measurement procedure [15]. The existence of interactions between the galloyl ring of the wine polyphenols that might be adsorbed onto the oral mucosa with the aromatic ring of the aroma molecules through π–π interactions could explain these results [12,17]. Results from the present study also confirm this explanation for other polar volatile molecules from the furanic chemical group (furfural, methyl furfural, and furfuryl alcohol), all of them characterized by the presence of an aromatic ring in their structure.

Regarding terpenes (limonene and α-pinene), which are characterized for being compounds with a high hydrophobicity (logP = 4.27 and 4.83, respectively) (Table 1), they showed very similar %OAR over the three sampling points, and therefore a high oral persistence. Only about 12% of the initial aroma amount was lost in the third in-mouth extraction (T3), and then 120 s after wine expectoration (Figure 2). As previously explained for the larger and more hydrophobic esters, stronger interactions between the most hydrophobic aroma compounds and the hydrophobic domains of the salivary mucins from the mucosal pellicle that covers the oral surface can be expected [28]. This might explain the higher oral aroma persistence observed for these wine aroma compounds.

On the other hand, the C_13_ norisoprenoid β-ionone, which also presented a high hydrophobicity (logP = 4.42) (Table 1), showed minor changes in the %OARt2 (10% of aroma loss compared to the first sampling point), but its oral release decreased almost 50% in the third sampling point (Figure 2). In previous works and using different methodologies such as intra-oral SPME [15] and in-nose PTR–MS [20], this compound showed a high oral persistence exhibiting relatively low oral aroma losses (around 40%) even at 4 min after the oral exposure to the wine.

Finally, in the case of the wine lactones (γ-butyrolactone and γ-nonalactone), they also showed minor changes in their oral release over the three sampling points with discrete aroma losses lower than 20% in both T2 and T3 sampling points (Figure 2). Despite the very different polarities of both lactones, γ-butyrolactone logP = −0.31 and γ-nonalactone logP = 2.08, their behavior was quite similar in the mouth, characterized by a high in-mouth persistence (Figure 2). The formation of Schiff bases between ketones and oral proteins (mucins, α-amylase) [2] might favor the higher adsorption of these compounds to the oral mucosa, thus being responsible for their higher oral persistence. However, this behavior should be confirmed in new in-vivo studies using synthetic wines supplemented with different types of ketone compounds with linear and cyclic structures.

### 3.2. Relationship between Oral Aroma Release and the Physicochemical Properties of Aroma Compounds

**Figure 3:**
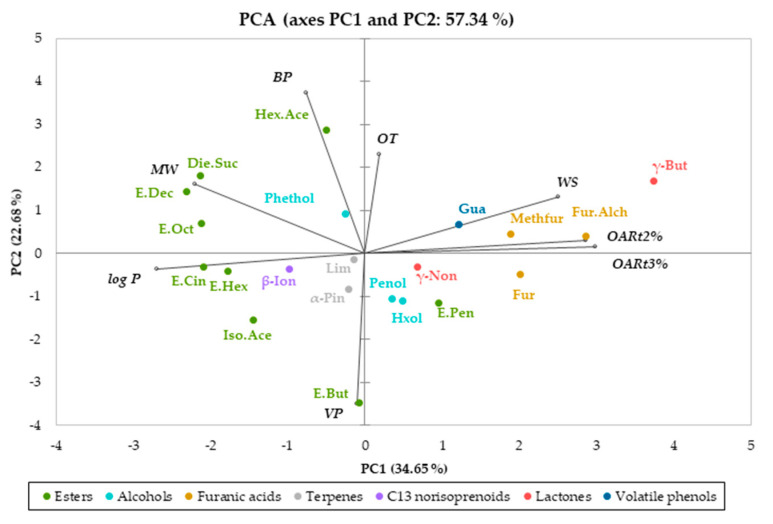
Principal component analysis (PCA) performed with some physicochemical features of the aroma compounds (boiling point (BP), hydrophobic constant (logP), odor threshold (OT)) and %OARt2 and %OARt3. logP: hydrophobic constant; BP: boiling point; OT: odor threshold; α-Pin: α-pinene; β-ion: β-ionone; γ-But: γ-butyrolactone; γ-Non: γ-nonalactone; Die.Suc: diethyl succinate; E.But: ethyl butanoate; E.Cin: ethyl cinnamate; E. Dec: ethyl decanoate; E.Hex: ethyl hexanoate; E. Oct: ethyl octanoate; E.Pen: ethyl pentanoate; Fur: furfural; Fur.Alc: furfuryl alcohol; Gua: guaiacol; Hex.Ace: hexyl acetate; Hxol: hexanol; Iso.Ace: isoamyl acetate; Lim: limonene; Methfur: 5-methyl furfural; Phethol: phenylethanol; Pntol: pentanol.

To further understand the relationship between the physicochemical properties of aroma compounds and the oral release behavior, we performed a principal component analysis (PCA). For this, some physicochemical properties such as hydrophobicity (logP) and boiling point, as well as other features such as the odor thresholds of the 22 wine aroma compounds identified in the mouth after wine rinsing (Table 1), were selected as independent variables. Additionally, the %OAR calculated after 60 (%OARt2) and 120 s (%OARt3) were used. The compound cis-3-hexen-1-ol was removed from this treatment, since, as previously shown, it showed atypical oral release behavior that deserves additional studies. Figure 3 shows the representation of this PCA.

As can be seen in Figure 3, the PCA biplot explained 70.43% of the total data variation. The first principal component (PC1) explained 43.49%, while the second one (PC2) explained 26.94% of the total variability. PC1 was positively correlated to the percentage of oral aroma release from both the second (%OARt2) and third (%OARt3) in-mouth sampling points. As can be seen, PC1 mainly separated the compounds according to their chemical family. The furanic group, guaiacol, the two lactones, and the two terpenes exhibited positive values for PC1, with the furanic group showing the highest factor loadings (0.8–0.9). On the contrary, the ester group (except ethyl pentanoate and hexyl acetate) and the alcohol group (βphenylethanol, hexanol, pentanol) were negatively related to this component. This confirms that the functional group is one of the main determinants of the oral release behavior, as previously suggested [4].

Additionally, PC1 was negatively correlated to the boiling point (BP) and hydrophobicity (logP) values. These results suggest that compounds with lower hydrophobicity (lower logP values), such as the furanic group, would show a higher oral aroma persistence compared to higher hydrophobic compounds. This could be because polar compounds are more easily dissolved in the water phase that covers the mucosal pellicle, which would produce an increase in their oral persistence, as recently suggested by Ployon and coworkers [28] using an ex vivo model. Thus, furanic acids (furfural, methyl furfural, and furfuryl alcohol), which were more polar in terms of logP (logP = 0.83, 0.67, and 0.45, respectively) (Table 1), were the compounds that showed the highest oral release in T2 and T3, exhibiting %OAR values higher than 95% in both sampling points (T2 and T3). However, this explanation does not seem valid for other polar compounds, such as the alcohols (with lower polarities compared to furanic compounds), in which the oral aroma persistence was relatively low (above 60% in both sampling points) compared to the furanic group. However, as previously shown, compound structure and the presence of aromatic rings in their molecule, as it is the case for some polar compounds (guaicol and furanic group), strongly determined their oral release behavior. In the same sense, the two compounds belonging to the terpene group, which have the highest logP values (4.27 and 4.83 for α-pinene and limonene, respectively), also showed higher oral aroma persistence compared to the alcohol group. This seems to corroborate the strong effect of the chemical structure on oral aroma persistence.

In the case of PC2, as shown in Figure 3, it practically did not show any correlation to the %OAR t2 and %OAR t3.

Nonetheless, it is also worth mentioning that the PCA results did not explain 100% of the data variation, which means that the physicochemical properties of the aroma compounds considered in this study were not the unique factor when explaining the oral aroma behavior of wine odorants after wine tasting. Other physicochemical features related to the molecular structure and the characteristics and composition of the oral environment might help explain the differences in oral aroma persistence among wine volatiles.

## 4. Conclusions

For the first time, using a large group of volunteers (*n* = 32) and a real wine, we determined the oral release behavior of 22 wine aroma compounds in the mouth at three different points after wine tasting (immediate, 60 s, and 120 s) by using a previously validated in-mouth HSSE procedure. Even considering the large inter-individual variations as a consequence of physiological features, some interesting trends in oral release behavior were observed depending on the chemical compound. In general, esters and linear alcohols showed the highest variations (large decrease) in oral aroma release, and therefore a low oral aroma persistence. On the contrary, terpenes, lactones, and C_13_ norisoprenoids showed lower variations in oral aroma release over the three sampling points, and therefore a higher oral aroma persistence. Additionally, and despite their low polarity, furanic acids and guaiacol showed the highest oral persistence. The oral release behavior was not only dependent on the physicochemical properties of the aroma compounds but also because of other features such as the molecular structure and probably on the characteristics and composition of the oral environment.

## Figures and Tables

**Table 1:** Retention times, physicochemical properties, and oral release (average values of three repetitions of absolute peak areas) of the 22 aroma compounds detected in the mouth at three different sampling points: 0 (T1), 60 (T2), and 120 (T3) seconds after wine expectoration. The table also shows the results from one-way ANOVA to check for significant differences (*p* < 0.05) between the three extraction times.

No. Compound	Aroma Compound	Retention Time (min)	Physicochemical Properties of Aroma Compounds						Oral Aroma Release (APAs) Over the Three Sampling Points			
			MW ^a^	logP ^a^	BP ^a^	VP ^a^	WS ^a^	OT ^b^	T1	T2	T3	Pr > F
	Esters											
1	Isoamyl acetate	7.46	130.19	2.26	142.5	5.67	1100	30 ^1^	2,495,178	963,196	524,939	<0.0001
2	Ethyl butanoate	5.53	116.16	1.85	121.5	14.6	2745	125 ^1^	317,302	301,709	258,753	0.681
3	Ethyl pentanoate	7.83	130.19	2.34	146.1	4.8	925.5	>200 ^2^	96,840	152,040	141,734	0.369
4	Ethyl hexanoate	10.52	144.22	2.83	167	1.8	308.7	62 ^1^	599,957	260,843	166,670	<0.0001
5	Ethyl octanoate	17.07	172.27	3.81	208.5	0.235	33.39	5 ^1^	492,678	226,935	151,675	<0.0001
6	Ethyl decanoate	23.23	200.32	4.79	241.5	0.0428	3517	200 ^1^	115,138	60,170	49,934	<0.0001
7	Hexyl acetate	11.97	144.22	2.83	171.5	1.45	308.7	1.5 ^3^	62,829	42,389	32,037	0.002
8	Diethyl succinate	24.09	174.2	1.39	217.7	0.147	5547	200,000 ^4^	257,106	112,972	66,785	<0.0001
9	Ethyl cinnamate	31.05	176.22	2.99	271	0.00874	160.6	5.1 ^1^	509,575	30,132	16,649	0.012
	*Alcohols*							-				
10	1-Pentanol	10.01	88.15	1.33	137.9	2.65	20,890	-	7,468,430	5,012,622	3,629,045	<0.0001
11	1-Hexanol	14.58	102.17	2.03	157.6	0.881	6885	8000 ^4^	522,716	308,090	210,761	<0.0001
12	Phenylethyl ethanol	28.54	122.17	1.57	218.2	0.0243	21,990	14,000 ^1^	2,289,276	1,332,593	994,164	0.000
13	Z-3-Hexen-1-ol	15.52	100.16	1.61	156.5	0.937	15,475	400 ^1^	59,232	117,719	116,501	0.163
	*Furanic acids*											
14	Furfural	17.78	96.09	0.83	161.7	2.32	53,580	14,100 ^1^	626,770	596,750	639,977	0.877
15	5-Methyl furfural	20.42	110.11	0.67	187	0.644	29,110	20,000 ^4^	111,669	103,936	103,787	0.821
16	Furfuryl alcohol	23.68	98.1	0.45	171	0.409	221,000	2000 ^4^	596,677	568,836	744,321	0.524
	*Terpenes*											
17	α-Pinene	5.25	136.24	4.27	155.5	4.02	34,834	-	278,068	244,054	248,766	0.961
18	Limonene	9.49	136.24	4.83	178	1.45	4.581	15 ^2^	435,510	347,603	387,864	0.721
	*C13 norisoprenoids*											
19	β-Ionone	29.3	192.3	4.42	127	0.0227	25.16	0.09 ^1^	783,236	83,581	33,204	0.017
	*Lactones*											
20	γ-Butyrolactone	22.3	86.09	-0.31	204	0.295	447,500	35,000 ^4^	128,301	104,228	101,701	0.304
21	γ-Nonalactone	30.1	156.23	2.08	136	0.0118	228.66	30 ^1^	98,120	65,455	56,939	0.039
	*Volatile phenols*											
22	Guaiacol	27.67	124.14	1.34	205	0.113	28,462	10 ^1^	91,729	74,640	78,168	0.106

^a^ MW: molecular weight; logP: hydrophobic constant; BP: boiling point; VP: vapor pressure; WS: water solubility. Parameters estimated using the software EPI Suite 4.11-Estimation Program Interface (U.S. EPA 2012). ^b^ OT: odor threshold calculated in hydroalcoholic and/or synthetic wines. ^1^ [33]; ^2^ [34]; ^3^ [35]; ^4^ [36].

## Supplementary Materials

The following are available online at https://www.mdpi.com/2304-8158/10/2/415/s1: Table S1: Volatile composition (average ± standard deviation in µg/L) of the red wine determined by HSSE-GC–MS and regression lines used for the quantification of aroma compounds in the wine. Figure S1. Chromatograms corresponding to the aroma profile of the wine (a) using HSSE and from the headspace of the mouth after spitting of the same wine using in-mouth HSSE (b). Numbers correspond to the compounds described in Table 1.
