# Supplementary material for: Oral Release Behavior of Wine Aroma Compounds by Using In-Mouth Headspace Sorptive Extraction (HSSE) Method
Source: Foods. 2021 Feb 13;10(2):415. doi: 10.3390/foods10020415 (PMC7918325; doi:10.3390/foods10020415)
Supplement: Supplementary file 1 [file foods-10-00415-s001.pdf]

Table S1. Volatile composition (average ± standard deviation in μg/L) of the red wine determined by headspace sorptive extraction Gas chromatography-Mass Spectrometry (HSSE-GC–MS) and regression lines used for the quantification of the aroma compounds in the wine.

Aroma Compound	Linear Range (μg/L)	Calibration Curve	R^2	Concentration in Wine (μg/L)
Esters				
Isoamyl acetate	0–2470	y = 9.6938x	0.98	3450 ± 230
Ethyl butanoate	0–2920	y = 4.8792x	0.99	217.63 ± 7.87
Ethyl pentanoate	0–2830	y = 6.4138x	0.98	116.55 ± 3.95
Ethyl hexanoate	0–1690	y = 7.5428x	0.98	777.71 ± 54.20
Ethyl octanoate	0–847	y = 7.7359x	0.97	215.52 ± 32.30
Ethyl decanoate	0–1290	y = 10.968x	0.97	162.23 ± 11.05
Hexyl acetate	0–1930	y = 9.5135x	0.98	81.81 ± 3.40
Diethyl succinate	0–4500	y = 0.2199x	0.97	5.56 ± 1.77
Ethyl cinnamate	0–2300	y = 1.825x	0.98	0.53 ± 0.04
Alcohols				
Pentanol	0–1620	y = 0.2003x	0.99	138.75 ± 11.96
Hexanol	0–3080	y = 0.8479x	0.98	49.17 ± 6.13
Phenylethanol	0–2450	y = 0.1382x	0.88	26.05 ± 10.54
Z-3-Hexen-1-ol	0–1990	y = 0.2613x	0.97	6.87 ± 0.68
Furanic acids				
Furfural	0–2990	y = 0.2579x	0.99	4.33 ± 0.63
5-methylfurfural	0–1550	y = 0.324x	0.98	0.94 ± 0.02
Furfuryl alcohol	0–4200	y = 0.0398x	0.93	0.23 ± 0.01
Terpenes				
α-Pinene	0–640	y = 23.037x	0.95	200.53 ± 7.87
Limonene	0–431	y = 12.576x	0.95	46.91 ± 4.22
C13 norisoprenoids				
β-Ionone	0–980	y = 5.2952x	0.99	3.82 ± 0.31
Lactones				
γ-Butyrolactone	0–8000	y = 0.0039x	0.62	0.01 ± 0.00
γ-Nonalactone	0–770	y = 0.582x	0.8	0.83 ± 0.28
Volatile phenols				
Guaiacol	0–1320	y = 0.4396x	0.96	0.45 ± 0.06

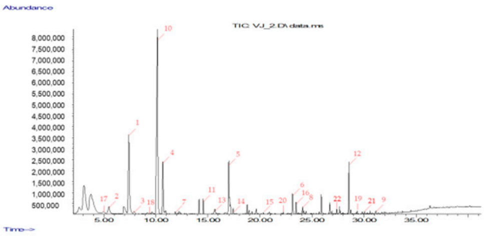

(**a**)

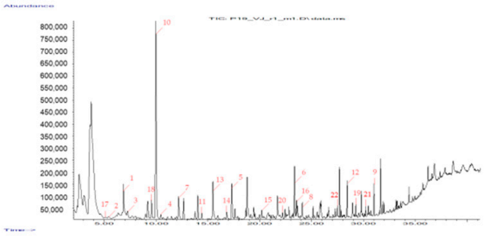

(**b**)

Figure S1. Chromatograms corresponding to the aroma profile of the wine (**a**) using HSSE and from the headspace of the mouth after spitting of the same wine using in-mouth HSSE (**b**). Numbers correspond to the compounds described in Table 1.
